# 4427 Angiopoietin F-domain valency determines outcome of Tie2 receptor engagement and accelerates angiogenesis in tissue regeneration

**DOI:** 10.1017/cts.2020.53

**Published:** 2020-07-29

**Authors:** Yan Ting Zhao, Jorge Fallas, Shally Saini, George Ueda, Logeshwaran Somasundaram, Ziben Zhou, Drew Seller, David Baker, Hannele Ruohola-Baker

**Affiliations:** 1 University of Washington

## Abstract

OBJECTIVES/GOALS: Lack of blood vessels remains a major obstacle in tissue regeneration. Angiopoietin 1 and 2 modulate angiogenesis through the Tie2 receptor tyrosine kinase. Ang1 activates pAKT to promote endothelial cell survival while Ang2 antagonizes these effects. We aim to dissect the Ang/Tie2 pathway to uncover the molecular basis for these opposing effects. METHODS/STUDY POPULATION: Ang1 and Ang2 bind Tie2 via nearly identical F-domains (Fd). To investigate the molecular basis regulating the Tie2 pathway, we generated a series of computationally designed self-assembling protein scaffolds presenting F-domains in a wide range of valencies and geometries using Rosette Molecular Modeling Suite. We examined the protein kinase activation, cell migration, and blood vessel formation produced by the designed proteins in human umbilical vein endothelial cells. RESULTS/ANTICIPATED RESULTS: Two phenotypic classes were demonstrated by the number of presented F domains: scaffolds presenting 3 or 4 Fd have Ang2 like activity, upregulating pFAK and pERK but not pAKT and failing to induce cell migration and tube formation; scaffolds presenting more than 6 Fd have Ang1 like activity, upregulating the three signaling branches and enhancing cell migration and tube formation. Scaffolds with 8 or more Fd show superagonist activity, producing significantly stronger phenotypes than Ang1. These results suggest that Fd valency largely determines Ang1 vs Ang2 signaling outcomes, and our designed superagonists can outperform Ang1 in vascularization and wound healing. In *in vivo* experiments, nanoparticles displaying 60 copies of Fd produce significant revascularization in hemorrhagic brains. DISCUSSION/SIGNIFICANCE OF IMPACT: Targeting the Tie2 pathway is a new paradigm in regenerative medicine. Our designed constructs will enable us to generate high-affinity Tie2 agonists and antagonists as drugs to control angiogenesis, enabling tissue regeneration that recapitulates the biological architecture of the native tissue physiology, improving organ transplant outcome.

